# Variation in Trust in Cancer Information Sources by Perceptions of Social Media Health Mis- and Disinformation and by Race and Ethnicity Among Adults in the United States: Cross-Sectional Study

**DOI:** 10.2196/54162

**Published:** 2024-05-08

**Authors:** Jim P Stimpson, Sungchul Park, Sandi L Pruitt, Alexander N Ortega

**Affiliations:** 1 Peter O’Donnell Jr. School of Public Health University of Texas Southwestern Medical Center Dallas, TX United States; 2 Department of Health Policy and Management Korea University Seoul Republic of Korea; 3 Thompson School of Social Work and Public Health University of Hawaiʻi at Mānoa Honolulu, HI United States

**Keywords:** cancer, United States, cross-sectional study, trust, consumer health information, misinformation, disinformation, race, ethnicity, cancer information, source, sources, perception, perceptions, social media, health information, cross-sectional data, misleading

## Abstract

**Background:**

Mis- and disinformation on social media have become widespread, which can lead to a lack of trust in health information sources and, in turn, lead to negative health outcomes. Moreover, the effect of mis- and disinformation on trust in information sources may vary by racial and ethnic minoritized populations.

**Objective:**

We evaluated how trust in multiple sources of cancer information varied by perceptions of health mis- and disinformation on social media and by race and ethnicity.

**Methods:**

Cross-sectional, nationally representative survey data from noninstitutionalized adults in the United States from the 2022 Health Information National Trends Survey 6 (HINTS 6) were analyzed (N=4137). The dependent variable measured the level of trust in cancer information sources. The independent variables were perceptions about health mis- and disinformation on social media and race and ethnicity. Multivariable logistic regression models were adjusted for survey weight and design, age, birth gender, race and ethnicity, marital status, urban/rural designation, education, employment status, feelings about household income, frequency of social media visits, and personal and family history of cancer. We also tested the interaction effect between perceptions of social media health mis- and disinformation and participants’ self-reported race and ethnicity.

**Results:**

Perception of “a lot of” health mis- and disinformation on social media, relative to perception of “less than a lot,” was associated with a lower likelihood of high levels of trusting cancer information from government health agencies (odds ratio [OR] 0.60, 95% CI 0.47-0.77), family or friends (OR 0.56, 95% CI 0.44-0.71), charitable organizations (OR 0.78, 95% CI 0.63-0.96), and religious organizations and leaders (OR 0.64, 95% CI 0.52-0.79). Among White participants, those who perceived a lot of health mis- and disinformation on social media were less likely to have high trust in cancer information from government health agencies (margin=61%, 95% CI 57%-66%) and family or friends (margin=49%, 95% CI 43%-55%) compared to those who perceived less than a lot of health mis- and disinformation on social media. Among Black participants, those who perceived a lot of health mis- and disinformation on social media were less likely to have high trust in cancer information from religious organizations and leaders (margin=20%, 95% CI 10%-30%) compared to participants who perceived no or a little health mis- and disinformation on social media.

**Conclusions:**

Certain sources of cancer information may need enhanced support against the threat of mis- and disinformation, such as government health agencies, charitable organizations, religious organizations and leaders, and family or friends. Moreover, interventions should partner with racial and ethnically minoritized populations that are more likely to have low trust in certain cancer information sources associated with mis- and disinformation on social media.

## Introduction

Misinformation is unintentionally providing false or inaccurate information, while disinformation is intentionally spreading false or inaccurate information [1-3]. A recent systematic review found that more than 80% of adult social media users perceive “some” or “a lot of” false or misleading health information on social media, while nearly a fifth reported either “none” or “a little” [4]. Both mis- and disinformation have been linked to reductions in health-promoting behaviors. For example, people who perceive more misinformation in the media are associated with a lower likelihood of being vaccinated against COVID-19 and a greater likelihood of smoking more and having poorer nutrition than people who perceive less misinformation in the media [5-11]. According to the Comprehensive Model of Information Seeking, misinformation may be associated with a lack of trust in health information sources, which can, in turn, lead to changes in health behaviors [12,13].

There is limited research on misinformation and trust, with some mixed findings. Some cross-sectional studies have found that higher perceptions of misinformation are associated with lower trust in the media, while one study of multiple countries, including the United States, did not find a relationship between perceptions of misinformation and trust in news media [14-19]. A gap in the literature is that these studies were not drawn from representative samples and only measured trust in media. Furthermore, the effects of misinformation may be more pronounced among individuals with comorbidities, particularly cancer, that have complex clinical treatment plans and significant economic costs [20]. For example, cancer survivors are more likely to have a lot of trust in information from doctors compared to persons that have not been diagnosed with cancer [20]. Therefore, there is an evidence gap for the effects of social media mis- and disinformation on trust in different credible sources (eg, scientists, doctors, and government health agencies) of cancer information.

The effect of mis- and disinformation on trust may also vary by different population groups. In some studies, racial and ethnic minoritized populations were found to be less likely to perceive false or misleading health information on social media and to trust noncredible information sources compared to non-Latino White people [21,22]. The lack of trust may also extend to credible sources of cancer information because, for example, non-Latino Black and Latino people have reported lower trust in doctors compared to non-Latino White people [20,23]. A study of 10-year trends in trust in cancer information found that, compared to non-Latino White participants, non-Latino Black participants were more likely to trust cancer information from media, government, charitable organizations, and religious organizations. In contrast, that same study found that Latino participants were less likely to trust cancer information from doctors compared to non-Latino White people [24]. There may be differences within Latino populations in trust in cancer information. For example, Cuban Americans and Puerto Ricans were more than twice as likely to trust information about cancer from print media and religious organizations compared to Mexican Americans [25]. However, a recent study found that trust in cancer information from government health agencies and family or friends declined among non-Latino Black participants from 2018 to 2020 [26]. Given these mixed findings, there is a need to examine whether the effect of mis- and disinformation on trust in cancer information varies among racial and ethnic minoritized populations and therefore may be a mechanism to explain these variations and a possible target for interventions to improve trust in cancer information, at least from credible sources such as doctors and scientists [27].

### Research Objective

The purpose of this study is to use recently released nationally representative data to estimate the association between perceptions of health information on social media and level of trust in multiple sources of information about cancer. We hypothesized that perception of a lot of health mis- and disinformation on social media would be associated with lower levels of trust in cancer information sources. By extension, this study evaluated the interaction effect between race and ethnicity of the participants, perceptions of social media health mis- and disinformation, and trust in cancer information. We hypothesized that the association between perceptions of a lot of mis- and disinformation on social media and trust in cancer information sources would vary by race and ethnicity. The results of this research have implications for effective communication about cancer in public health education campaigns, especially for racial and ethnic minoritized populations.

## Methods

### Data

This study used cross-sectional data from the Health Information National Trends Survey 6 (HINTS 6), which is a nationally representative survey of civilian, noninstitutionalized adults aged 18 years and older living in the United States. HINTS 6 provides data on adults’ knowledge of cancer risk factors, attitudes toward cancer screening, and cancer prevention and screening behaviors. HINTS 6 used a 2-stage probability sample of residential addresses. Mail and online surveys were administered to household members from March 7 to November 8, 2022, with a response rate of 28.1% [28]. The data are publicly available and deidentified. Further details about the survey methodology and recruitment procedures are available from the HINTS 6 Methodology Report [28].

Given the focus of this study was perceptions of false or misleading health information on social media, persons that reported that they did not use social media were excluded. There were 4710 cases with complete data for the dependent and independent variables. After using listwise deletion for 573 cases with missing data for the control variables, the final analytical sample consisted of 4137 adult social media users.

### Measures

Our dependent variables were measured by asking participants, “In general, how much would you trust information about cancer from...” Responses included the following: “a doctor,” “family or friends,” “religious organizations and leaders,” “government health agencies,” “charitable organizations,” and “scientists.” The response options were dichotomized into low levels of trust (“not at all” or “a little”) versus high levels of trust (“some” or “a lot”).

The primary independent variable was perceptions about health mis- and disinformation on social media, which was assessed by the following question: “How much of the health information that you see on social media do you think is false or misleading?” HINTS had not measured perceptions about social media mis- and disinformation in prior iterations of the survey. However, this measure did not differentiate between people’s perceptions of mis- versus disinformation. The original response categories were “a lot,” “some,” “a little,” and “none.” We dichotomized this as “less than a lot” (including “some,” “a little,” and “ none”) versus “a lot.” Race and ethnicity were self-reported by the participants in 5 categories: “non-Latino White,” “non-Latino Black,” “Asian American,” “other,” and “Latino.”

Demographic control variables included age (18-34, 35-49, 50-64, and ≥65 years), sex (male and female), marital status (married or cohabiting, formerly married, and never married), residence in a metropolitan versus nonmetropolitan county as designated by the United States Department of Agriculture in 2013, education (high school or less, some college, and college degree or higher), full-time employment status, and feelings about household income (finding it very difficult on present income, getting by on present income, and living comfortably on present income). It should be noted that age was not collected as a continuous variable in HINTS 6, which limited the age categories that could be analyzed. In addition, we controlled for frequency of visiting social media sites (never, monthly/weekly, and daily) in the past 12 months and personal and family (first- or second-degree biological relatives) history of cancer.

### Statistical Analysis

All analyses accounted for survey weights and design using jackknife replicate weights for variance estimation. Statistical significance was set at α<.05. The descriptive statistics for the study sample were calculated as survey-weighted percentages accompanied with the raw sample size for each variable. The bivariate relationship between level of trust in cancer information and perceptions of mis- and disinformation were calculated with cell percentages and adjusted Wald *P* values. Then, multivariable logistic regression models were calculated for each dichotomous outcome. In addition to the main effect, we also tested the interaction effect between perceptions of health mis- and disinformation on social media and participants’ self-reported race and ethnicity. To facilitate interpretation of the interaction effect, we calculated predicted marginal effects from the multivariable logistic regression models.

For this study, the primary focus was perceptions of information on social media. Therefore, we conducted a sensitivity analysis in which we excluded 257 adults who had not visited a social media site in the past year or reported that they did not use social media (n=3880 were included). After excluding these participants, the results were similar, as shown in Multimedia Appendix 1, Table S1. In addition, we conducted a sensitivity analysis for an ordinal measurement of the dependent variables (“a lot,” “some,” “a little,” and “not at all”) using ordered logit regression, and we found that the results were replicated with this alternative measurement, as shown in Multimedia Appendix 1, Table S2. Another sensitivity analysis included participants that did not use social media (n=4986). After including participants that did not use social media, the results were similar, as shown in Multimedia Appendix 1, Table S3. In Multimedia Appendix 1, Table S4, we tested an alternative measurement of the independent variable in which perception of “a lot” of social media mis- and disinformation was compared with respondents that reported “some” and “none” or “a little.” For this sensitivity analysis, we combined “none” and “a little” because only 108 participants chose “none” for this measure. We replicated the main result using this alternative measurement of the independent variable.

### Ethical Considerations

The University of Texas Southwestern Medical Center institutional review board determined that the study was exempt from review because it used publicly available data without personal identifiers.

## Results

Table 1 provides the survey-weighted percentages for the study variables. Most participants in the survey reported high trust in cancer information from doctors (95%), scientists (86%), and government health agencies (71%). About half reported high trust in cancer information from family or friends (54%) and charitable organizations (49%). About a quarter of participants reported high trust in cancer information from religious organizations and leaders (26%). When participants were asked about perceptions of false or misleading health information on social media, most reported “less than a lot” (63%) and 37% reported “a lot.”

**Table 1:** Unadjusted sample size and survey-weighted percentages for study variables from the 2022 Health Information National Trends Survey 6 (N=4137).

Variables				Unadjusted sample size, n (weighted %)
**Outcome variables**				
	**In general, how much would you trust information about cancer from a doctor?**			
		Low	200 (5)	
		High	3937 (95)	
	**In general, how much would you trust information about cancer from scientists?**			
		Low	525 (14)	
		High	3612 (86)	
	**In general, how much would you trust information about cancer from government health agencies?**			
		Low	1077 (29)	
		High	3060 (71)	
	**In general, how much would you trust information about cancer from family or friends?**			
		Low	1874 (46)	
		High	2263 (54)	
	**In general, how much would you trust information about cancer from charitable organizations?**			
		Low	2050 (51)	
		High	2087 (49)	
	**In general, how much would you trust information about cancer from religious organizations and leaders?**			
		Low	3034 (74)	
		High	1103 (26)	
**Independent variables**				
	**How much of the health information that you see on social media do you think is false or misleading?**			
		Less than a lot	2643 (63)	
		A lot	1494 (37)	
	**Race and ethnicity**			
		Non-Latino White	2381 (61)	
		Non-Latino Black	643 (11)	
		Latino	734 (17)	
		Non-Latino Asian American	230 (6)	
		Non-Latino other	149 (5)	
	**Age group (years)**			
		18-34	771 (29)	
		35-49	1012 (29)	
		50-64	1222 (27)	
		≥65	1132 (15)	
	**Birth gender**			
		Male	1586 (48)	
		Female	2551 (52)	
	**Marital status**			
		Married/cohabiting	2290 (57)	
		Formerly married	994 (10)	
		Never married	853 (33)	
	**United States Department of Agriculture 2013 rural/urban designation**			
		Nonmetropolitan	512 (12)	
		Metropolitan	3625 (88)	
	**Education**			
		High school or less	812 (25)	
		Some college	1185 (39)	
		College graduate or higher	2140 (36)	
	**Work full time (past 30 days)**			
		No	1878 (40)	
		Yes	2259 (60)	
	**Feelings about household income**			
		Finding it very difficult on present income	811 (19)	
		Getting by on present income	1505 (36)	
		Living comfortably on present income	1821 (45)	
	**Frequency of social media site visits**			
		Never	257 (6)	
		Monthly or weekly	1119 (25)	
		Daily	2761 (70)	
	**Personal history of cancer**			
		No	3593 (91)	
		Yes	544 (9)	
	**Family history of cancer**			
		No	1259 (35)	
		Yes	2878 (65)	

Table 2 provides the bivariable relationship between the outcome variables and the independent variable. There was not a statistically significant relationship between perception of mis- and disinformation and trust in cancer information from doctors (*P*=.93) or scientists (*P*=.85). However, there was a statistically significant bivariable relationship between perception of mis- and disinformation and trust in cancer information from government health agencies (*P*<.001), family or friends (*P*<.001), charitable organizations (*P*=.007), and religious organizations and leaders (*P*<.001). About a quarter of participants (24%) that perceived a lot of mis- and disinformation on social media had a high level of trust in government health agencies. Nearly half of participants (47%) that perceived less than a lot of mis- and disinformation on social media had a high level of trust in government health agencies. Only 17% of participants that perceived a lot of mis- and disinformation on social media had a high level of trust in family or friends. In contrast, 37% of participants that perceived less than a lot of mis- and disinformation on social media had a high level of trust in family or friends. Only 16% of participants that perceived a lot of mis- and disinformation on social media had a high level of trust in charitable organizations. A third of participants (33%) that perceived less than a lot of mis- and disinformation on social media had a high level of trust in charitable organizations. Finally, only 7% of participants that perceived a lot of mis- and disinformation on social media had a high level of trust in religious organizations and leaders. Nearly 1 in 5 participants (19%) that perceived less than a lot of mis- and disinformation on social media had a high level of trust in religious organizations and leaders.

**Table 2:** Survey-weighted unadjusted bivariable relationship between trust in cancer information source (low vs high) and perception of health mis- and disinformation on social media (“less than a lot” vs “a lot”) from the 2022 Health Information National Trends Survey 6 (N=4137).

Cancer information source	Trust in cancer information source and perception of health mis- and disinformation on social media					*P* value^a^
	Low trust		High trust			
	Less than a lot^b^, %	A lot^c^, %	Less than a lot^b^, %	A lot^c^, %		
Doctors	3	2	60	35	.93	
Scientists	9	5	54	32	.85	
Government health agencies	16	13	47	24	<.001	
Family or friends	26	20	37	17	<.001	
Charitable organizations	31	20	33	16	.007	
Religious organizations and leaders	45	29	19	7	<.001	

^a^*P* values were calculated with the adjusted Wald *χ*^2^ test.

^b^Perception of “less than a lot” of health mis- and disinformation on social media.

^c^Perception of “a lot” of health mis- and disinformation on social media.

Table 3 provides the multivariable odds ratios (ORs) and 95% CIs calculated from logistic regression. Perception of a lot of health mis- and disinformation on social media, relative to perception of less than a lot, was associated with a lower likelihood of high levels of trusting cancer information from government health agencies (OR 0.60, 95% CI 0.47-0.77), family or friends (OR 0.56, 95% CI 0.44-0.71), charitable organizations (OR 0.78, 95% CI 0.63-0.96), and religious organizations and leaders (OR 0.64, 95% CI 0.52-0.79).There was not a statistically significant association between perception of social media health mis- and disinformation and level of trust in cancer information from doctors (OR 0.95, 95% CI 0.45-2.01) or scientists (OR 0.98, 95% CI 0.72-1.33).

**Table 3:** Multivariable odds ratios (ORs) and 95% CIs for perceptions of social media health mis- and disinformation and trust in cancer information sources from the 2022 Health Information National Trends Survey 6 (N=4137). Logistic regression models were adjusted for survey weight and design, age, birth gender, marital status, urban or rural designation, race and ethnicity, education, employment status, feelings about household income, frequency of social media visits, and personal and family history of cancer.

Cancer information source	Trust in cancer information source among participants with the perception that a lot of health information on social media is false or misleading, odds ratio^a^ (95% CI)	
Doctors	0.95 (0.45-2.01)	
Scientists	0.98 (0.72-1.33)	
Government health agencies	0.60 (0.47-0.77)	
Family or friends	0.56 (0.44-0.71)	
Charitable organizations	0.78 (0.63-0.96)	
Religious organizations and leaders	0.64 (0.52-0.79)	

^a^Reference: “less than a lot.”

Table 4 provides the predicted marginal effects, interpreted as percentage points, calculated from the multivariable logistic regression–adjusted interaction effects between perceptions of health mis- and disinformation on social media and participants’ self-reported race and ethnicity. There was not a statistically significant interaction effect between perception of mis- and disinformation, race and ethnicity, and trust in cancer information from doctors or scientists. Among White participants, those who perceived a lot of health misinformation and disinformation on social media were less likely to have high trust in cancer information from government health agencies (margin=61%, 95% CI 57%-66%) and family or friends (margin=49%, 95% CI 43%-55%) compared to those who perceived less than a lot of health mis- and disinformation on social media. Among Black participants, those who perceived a lot of health mis- and disinformation on social media were less likely to have high trust in cancer information from religious organizations and leaders (margin=20%, 95% CI 10%-30%) compared to participants who perceived less than a lot of health mis- and disinformation on social media.

**Table 4:** Multivariable-adjusted percentage points for trusting cancer information by source and the interaction effect between race and ethnicity and perceptions of health mis- and disinformation on social media from the 2022 Health Information National Trends Survey 6 (N=4137). Predicted marginal effects were calculated from multivariable logistic regression models that were adjusted for survey weight and design, age, birth gender, marital status, urban or rural designation, education, employment status, feelings about household income, frequency of social media visits, and personal and family history of cancer.

Race and ethnicity		Perception of false or misleading health information from cancer information source, percentage points (95% CI)					
		Doctors	Scientists	Government health agencies	Family or friends	Charitable organizations	Religious organizations and leaders
						
**Non-Latino White**							
	Less than a lot	96 (93-98)	86 (83-90)	74 (71-78)	62 (57-68)	51 (46-56)	23 (20-26)
	A lot	95 (93-97)	85 (82-89)	61 (57-66)	49 (43-55)	43 (38-49)	19 (15-22)
**Non-Latino Black**							
	Less than a lot	96 (94-99)	80 (71-88)	75 (66-84)	62 (53-71)	57 (50-64)	49 (40-57)
	A lot	96 (92-101)	86 (76-95)	71 (60-83)	47 (33-61)	58 (44-73)	20 (10-30)
**Latino**							
	Less than a lot	93 (89-98)	87 (83-92)	77 (68-85)	48 (39-56)	50 (42-58)	40 (28-51)
	A lot	96 (92-100)	87 (82-95)	72 (63-82)	37 (28-47)	57 (46-68)	29 (17-40)
**Non-Latino Asian American**							
	Less than a lot	89 (61-117)	90 (82-98)	77 (58-96)	54 (41-67)	44 (30-58)	26 (12-40)
	A lot	99 (93-105)	93 (80-104)	86 (68-103)	32 (14-50)	34 (9-59)	23 (4-42)
**Non-Latino Other**							
	Less than a lot	96 (84-107)	89 (78-100)	82 (72-92)	68 (53-82)	58 (38-77)	33 (16-50)
	A lot	85 (70-99)	86 (70-101)	56 (36-77)	46 (26-67)	28 (14-42)	13 (2-24)

## Discussion

### Principal Findings

We found that trust in cancer information from doctors or scientists did not vary based on perceptions of health mis- and disinformation on social media. This suggests that people view doctors and scientists as credible sources of cancer information. However, we found that perception of a lot of mis- and disinformation was associated with reduced levels of trust in cancer information from family or friends, government health agencies, charitable organizations, and religious organizations and leaders. This finding supports other studies that found that mis- and disinformation is associated with reductions in trust in media but extends this prior literature by finding an impact on trust in other sources of cancer information [14-19]. Moreover, this finding is consistent with the Comprehensive Model of Information Seeking, which identifies trust as a mechanism linking mis- and disinformation to health behaviors [12,13].

There were notable variations in the relationship between trust in cancer information sources, perceptions of false or misleading health information, and race and ethnicity. For instance, we found that Black participants who perceived a lot of health mis- and disinformation on social media were less likely to have high trust in cancer information from religious organizations and leaders compared to Black participants who perceived less than a lot of health mis- and disinformation on social media. Another contribution of our study is that White participants who perceived a lot of health mis- and disinformation on social media were less likely to have high trust in cancer information from government health agencies and family or friends compared to White participants who perceived less than a lot of health mis- and disinformation on social media. There have been mixed findings on trust in cancer information sources by race and ethnicity in the recent literature, with one study finding higher trust among Black participants for several sources of cancer information compared to White participants and lower trust in doctors among Latino participants compared to White participants [21-25]. However, another study found that trust in cancer information from government health agencies and family or friends declined among Black participants after the COVID-19 pandemic [26]. Our study adds to this literature by identifying that the effect of mis- and disinformation on trusting information sources may vary among racial and ethnic minoritized populations.

### Limitations

We were able to replicate the findings of the study using several different sensitivity analyses, as shown in Multimedia Appendix 1. However, the results should be interpreted within the constraints of the cross-sectional data. First, this study cannot be used to determine the causal relationship between perceptions of mis- and disinformation and trust in social institutions. Second, the 2022 wave of the HINTS survey was the first time that the public’s perceptions of mis- and disinformation were measured. If this measure is collected in subsequent iterations of HINTS, then analyses may be able to detect changes in the association between mis- and disinformation and trust in information sources over time. We note that perceptions of mis- and disinformation may not be an accurate measure of objective exposure to social media mis- and disinformation. Further, this measure does not differentiate between people’s perceptions of mis- versus disinformation. Another limitation is that the focus of this study was on social media mis- and disinformation rather than all media, such as traditional television and print, and therefore the results should be interpreted for this specific form of media. Finally, this study focused on trust in cancer information, and the findings might not apply to trust in other types of health information. By extension, levels of trust in government information may differ between federal and state government health agencies, which were not differentiated in our study [29,30].

### Conclusion

Certain sources of cancer information may need enhanced support from the threat of mis- and disinformation, such as government health agencies, charitable organizations, religious organizations and leaders, and family or friends. Moreover, there were notable variations in the relationship between trust in cancer information sources (government health agencies, family or friends, and religious organizations and leaders), perceptions of false or misleading health information, and race and ethnicity. One positive finding is that perceptions of mis- and disinformation were not associated with levels of trust in credible sources of cancer information such as doctors or scientists overall or by race and ethnicity. In prior work, researchers have suggested that interventions should be focused on improving trust in science [1]. Although bolstering trust in science or doctors is important, our findings indicate that other sources of cancer information may be more susceptible to the threat of mis- and disinformation. Moreover, interventions should partner with racial and ethnically minoritized populations that are more likely to have low trust in certain cancer information sources associated with mis- and disinformation on social media.

## Appendix

Multimedia Appendix 1 Supplemental analyses.

## Abbreviations

HINTS: Health Information National Trends Survey.
OR: odds ratio.

## Data Availability

The data sets generated during and/or analyzed during this study are available in the National Cancer Institute repository [31].
