# Supplementary material for: Variation in Trust in Cancer Information Sources by Perceptions of Social Media Health Mis- and Disinformation and by Race and Ethnicity Among Adults in the United States: Cross-Sectional Study
Source: JMIR Cancer. 2024 May 8;10:e54162. doi: 10.2196/54162 (PMC11112477; doi:10.2196/54162)
Supplement: Multimedia Appendix 1 [file cancer_v10i1e54162_app1.docx]

MULTIMEDIA APPENDIX

Table S1: Multivariable logistic regression models for trusting cancer information by source among social media users in the past year, HINTS 6, 2022, N = 3,880

|  | **Trust of Cancer Information Source** | | | | | | | | | | | |
| --- | --- | --- | --- | --- | --- | --- | --- | --- | --- | --- | --- | --- |
|  | **Doctor** | | **Scientists** | | **Government Health Agencies** | | **Family or Friends** | | **Charitable Organizations** | | **Religious Organizations & Leaders** | |
|  | **OR** | **95% CI** | **OR** | **95% CI** | **OR** | **95% CI** | **OR** | **95% CI** | **OR** | **95% CI** | **OR** | **95% CI** |
| Perception of amount of health information on social media that is false or misleading |  |  |  |  |  |  |  |  |  |  |  |  |
| < A lot (ref) |  |  |  |  |  |  |  |  |  |  |  |  |
| A lot | .84 | .49, 1.44 | 1.06 | .75, 1.49 | .61 | .48, .76 | .55 | .44, .70 | .78 | .62, .97 | .65 | .52, .80 |

Note: OR=Odds Ratio; CI = Confidence Interval. This logistic regression analysis excluded 257 adult social media users who had not visited a social media site in the past year.

Table S2: Multivariable ordered logit regression models for ordinal measurement of trusting cancer information by source, HINTS 6, 2022, N = 4,137

|  | **Trust of Cancer Information Source** | | | | | | | | | | | |
| --- | --- | --- | --- | --- | --- | --- | --- | --- | --- | --- | --- | --- |
|  | **Doctor** | | **Scientists** | | **Government Health Agencies** | | **Family or Friends** | | **Charitable Organizations** | | **Religious Organizations & Leaders** | |
|  | **OR** | **95% CI** | **OR** | **95% CI** | **OR** | **95% CI** | **OR** | **95% CI** | **OR** | **95% CI** | **OR** | **95% CI** |
| Perception of amount of health information on social media that is false or misleading |  |  |  |  |  |  |  |  |  |  |  |  |
| < A lot (ref) |  |  |  |  |  |  |  |  |  |  |  |  |
| A lot | .89 | .64, 1.23 | 1.07 | .86, 1.34 | .71 | .55, .91 | .56 | .45, .72 | .73 | .58, .92 | .56 | .46, .67 |

Note: OR=Odds Ratio; CI = Confidence Interval. This ordered logit regression analysis used an alternative measurement of the outcome variable coded as A lot, Some, A little, Not at All.

Table S3: Multivariable logistic regression models for trusting cancer information by source among all survey participants, HINTS 6, 2022, N = 4,986

|  | **Trust of Cancer Information Source** | | | | | | | | | | | |
| --- | --- | --- | --- | --- | --- | --- | --- | --- | --- | --- | --- | --- |
|  | **Doctor** | | **Scientists** | | **Government Health Agencies** | | **Family or Friends** | | **Charitable Organizations** | | **Religious Organizations & Leaders** | |
|  | **OR** | **95% CI** | **OR** | **95% CI** | **OR** | **95% CI** | **OR** | **95% CI** | **OR** | **95% CI** | **OR** | **95% CI** |
| Perception of amount of health information on social media that is false or misleading |  |  |  |  |  |  |  |  |  |  |  |  |
| < A lot (ref) |  |  |  |  |  |  |  |  |  |  |  |  |
| A lot | 1.17 | .72, 1.89 | .94 | .70, 1.27 | .69 | .53, .88 | .63 | .52, .76 | .73 | .58, .92 | .72 | .55, .93 |

Note: OR=Odds Ratio; CI = Confidence Interval. This logistic regression analysis included participants that do not use social media.

Table S4: Multivariable logistic regression models for trusting cancer information by source, HINTS 6, 2022, N = 4,137

|  | **Trust of Cancer Information Source** | | | | | | | | | | | |
| --- | --- | --- | --- | --- | --- | --- | --- | --- | --- | --- | --- | --- |
|  | **Doctor** | | **Scientists** | | **Government Health Agencies** | | **Family or Friends** | | **Charitable Organizations** | | **Religious Organizations & Leaders** | |
|  | **OR** | **95% CI** | **OR** | **95% CI** | **OR** | **95% CI** | **OR** | **95% CI** | **OR** | **95% CI** | **OR** | **95% CI** |
| Perception of amount of health information on social media that is false or misleading |  |  |  |  |  |  |  |  |  |  |  |  |
| None / A little (ref) |  |  |  |  |  |  |  |  |  |  |  |  |
| Some | 1.56 | .84, 2.91 | .95 | .61, 1.48 | .84 | .61, 1.17 | .82 | .63, 1.07 | .75 | .57, .99 | .78 | .54, 1.11 |
| A lot | 1.27 | .59, 2.71 | .95 | .62, 1.42 | .53 | .38, .73 | .49 | .38, .63 | .64 | .47, .86 | .53 | .39, .72 |

Note: OR=Odds Ratio; CI = Confidence Interval. In this logistic regression analysis, we tested an alternative measurement of the independent variable in which perception of ‘a lot’ of social media mis-disinformation was compared with respondents that reported ‘some’ and ‘none or a little.’ For this sensitivity analysis, we combined ‘none and a little’ because only 108 participants chose ‘none’ for this measure.
